# Transcriptome analysis of germinating maize kernels exposed to smoke-water and the active compound KAR_1_

**DOI:** 10.1186/1471-2229-10-236

**Published:** 2010-11-02

**Authors:** Vilmos Soós, Endre Sebestyén, Angéla Juhász, Marnie E Light, Ladislav Kohout, Gabriella Szalai, Júlia Tandori, Johannes Van Staden, Ervin Balázs

**Affiliations:** 1 Department of Applied Genomics, Agricultural Research Institute of the Hungarian Academy of Sciences, H-2462 Martonvásár, Brunszvik u. 2, Hungary; 2 Research Centre for Plant Growth and Development, School of Biological and Conservation Sciences, University of KwaZulu-Natal Pietermaritzburg, Private Bag X01, Scottsville 3209, South Africa; 3 Institute of Organic Chemistry and Biochemistry, Academy of Sciences of the Czech Republic, v.v.i., Flemingovo nám. 2, 166 10 Prague 6, Czech Republic; 4 Department of Plant Physiology, Agricultural Research Institute of the Hungarian Academy of Sciences, H-2462 Martonvásár, Brunszvik u. 2, Hungary

## Abstract

**Background:**

Smoke released from burning vegetation functions as an important environmental signal promoting the germination of many plant species following a fire. It not only promotes the germination of species from fire-prone habitats, but several species from non-fire-prone areas also respond, including some crops. The germination stimulatory activity can largely be attributed to the presence of a highly active butenolide compound, 3-methyl-2*H*-furo[2,3-*c*]pyran-2-one (referred to as karrikin 1 or KAR_1_), that has previously been isolated from plant-derived smoke. Several hypotheses have arisen regarding the molecular background of smoke and KAR_1 _action.

**Results:**

In this paper we demonstrate that although smoke-water and KAR_1 _treatment of maize kernels result in a similar physiological response, the gene expression and the protein ubiquitination patterns are quite different. Treatment with smoke-water enhanced the ubiquitination of proteins and activated protein-degradation-related genes. This effect was completely absent from KAR_1_-treated kernels, in which a specific aquaporin gene was distinctly upregulated.

**Conclusions:**

Our findings indicate that the array of bioactive compounds present in smoke-water form an environmental signal that may act together in germination stimulation. It is highly possible that the smoke/KAR_1 _'signal' is perceived by a receptor that is shared with the signal transduction system implied in perceiving environmental cues (especially stresses and light), or some kind of specialized receptor exists in fire-prone plant species which diverged from a more general one present in a common ancestor, and also found in non fire-prone plants allowing for a somewhat weaker but still significant response. Besides their obvious use in agricultural practices, smoke and KAR_1 _can be used in studies to gain further insight into the transcriptional changes during germination.

## Background

Smoke released by natural fires is a major environmental cue in fire-prone habitats and a wide range of species show enhanced germination responses after exposure to aerosol smoke or smoke-water. In addition, several species from non-fire prone regions, and some major crops respond to various smoke treatments. Smoke can also positively affect the post-germination stage resulting in increased seedling vigour [1]. Efforts to identify the active compound from smoke-water resulted in the characterization of 3-methyl-2*H*-furo[2,3-*c*]pyran-2-one using achenes of *Lactuca sativa *cv. Grand Rapids [2] or the seeds of *Conostylis aculeata *and *Stylidium affine *[3] as germination test systems. This butenolide-type compound promotes germination over a very wide range of concentrations, from 10^-4 ^M down to 10^-9 ^M, spanning five orders of magnitude [4], and the action of smoke in promoting the germination of seeds of many species is mainly attributed to the presence of this compound in smoke. Currently, at least five analogues of KAR_1 _(referred as KAR_2_-KAR_6 _[5]) can be found in smoke and some of these are likely to contribute to the overall germination promoting activity of smoke extracts. In addition, it was shown that 'dual regulatory' cues exist in the smoke which can either have promoting or inhibitory effects on germination [6,7]. The suspicion that inhibitory constituents are also present in the smoke was confirmed recently when a related butenolide, 3,4,5-trimethylfuran-2(5*H*)-one, was characterized from smoke showing an inhibitory effect on germination [8]. The study revealed that the action of the compound is concentration dependent and significantly reduces the effect of KAR_1 _(promoter) when lettuce achenes were treated simultaneously, irrespective of the KAR_1 _concentrations applied.

There is currently little knowledge on the molecular background of smoke- and KAR_1_-stimulated germination and the observed increase in seedling vigour. The studies published to date have typically been physiological in nature, investigating similarities between the effects of smoke and other plant growth regulators, such as gibberellins and strigolactones. Deeper insight into the molecular background of smoke action has been published more recently [1,9,10]. We reported that the application of smoke-water to maize kernels yielded seedlings with higher vigour and resulted in the induction of stress-related changes in the global transcriptome of young seedlings [1]. Thus, it appears that the 'hardening' effect of smoke is similar to that caused by abscisic acid (ABA). The chain of events in the transcriptome during imbibition, and the genes orchestrating the effect of smoke and KAR_1 _are still elusive. However, the identification of the active component in smoke (i.e. KAR_1_) presents enhanced opportunities for elucidating the mode of action of this compound in the absence of artefacts and confounding influences caused by the additional compounds in smoke.

It is well established that the application of smoke and KAR_1 _breaks seed dormancy and yields earlier testa rupture and overall higher germination rate, although these responses can vary between species. Thus, smoke and KAR_1 _treatments have the potential to improve not only the germination percentage but also the seedling vigour of many species. Regarding maize, this effect is more pronounced as smoke and KAR_1 _treatment results in a massive increase in post-germination growth and seedling vigour [1,11]. On the other hand, smoke and KAR_1 _positively affects the germination rate of maize, as determined by a general germination test, and slightly enhances the water uptake and imbibition of the kernels in the pre-germinative stage [1,11]. Other reports suggest that smoke and KAR_1 _affect initial water uptake in tomato [12] and water homeostasis during germination in *Eragrostis tef *[13].

Our previous microarray study on smoke-exposed maize seedlings showed that smoke treatment results in a distinct, although not robust, change in the gene expression pattern. The aim of the present study was to gain a deeper insight into the molecular background of how smoke and KAR_1 _exert their effects on seed germination during imbibition, prior to testa rupture. To elucidate the action of smoke-water and KAR_1 _in the early imbibition stages of maize germination, we recorded the changes in the total transcriptome in the first 24 h in a time-course microarray experiment. Here, we present a detailed comparative analysis of the changes in gene expression that take place in maize embryos after exposure to smoke-water and KAR_1_. The present work substantially extends our current knowledge of transcriptional regulation by smoke and KAR_1 _exposure and will provide valuable insight into which aspects of smoke- and KAR_1_-induced germination and increased seedling vigour should be the focus of further studies.

## Results

### Germination characteristics of smoke-water and KAR_1_-treated kernels

**Figure 1:**
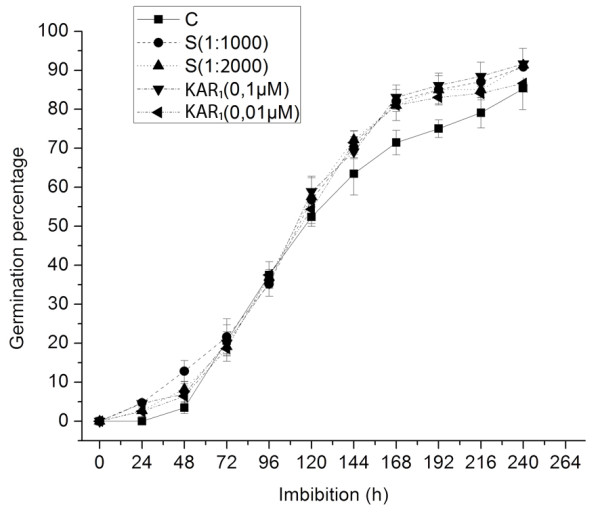
**Effect of smoke and KAR_1 _on the germination time course of maize kernels**. Each treatment consisted of four independent experiments with three biological replicates (*n *= 30). The kernels were treated with water (control), 1:1000 or 1:2000 (v/v) dilution of smoke-water, and 0.1 or 0.01 μM KAR_1_. Germinated kernels were scored every day for 10 d. Error bars represent standard error (SE) of the mean germination percentage.

Application of smoke-water and the active compound KAR_1 _slightly, but significantly, increased the germination rate of the treated kernels after 10 d, when water imbibed kernels were used as controls (Figure 1). The time course of testa rupture was similar in all conditions applied, however, the first appearance of radicles/coleoptiles was after about 24 h in the treated samples. The actual germination percentage was higher in the smoke- and KAR_1_-exposed kernels from 5 d onwards, and beyond 10 d, no further testa rupture was observed.

For experimental design reasons, we assumed that the effect of both cues on the germination is equivalent, regarding their apparent physiological effect on germination parameters. Considering the presence of inhibitory compound(s) in smoke, the concentration of which may be the limiting factor for the germination activity of smoke-water, the most suitable range for which germination promotion by smoke occurred was determined, and we found that the dilutions used in previous reports (1:1000 and 1:2000) worked well in our experiments. We also determined the concentration of KAR_1 _and 3,4,5-trimethylfuran-2(5*H*)-one in our smoke-water batches. The concentration of the KAR_1 _in crude smoke-water was 4.0818 × 10^-6 ^M ± 3.6% (0.004 μM in the diluted smoke-water), whereas the concentration of the inhibitory compound was 1.3 × 10^-2 ^M ± 5.8% in the undiluted smoke-water. The 3,4,5-trimethylfuran-2(5*H*)-one concentration was much higher in the crude smoke than the reported 10 μM limit, which is highly inhibitory to germination [8]. Therefore, to achieve a positive germination response we used the 1:1000 dilution of the smoke-water, a concentration applied in previous studies [1,6,7].

Taking into account that KAR_1 _is active over a very wide concentration range, the concentration of the inhibitory compound is the limiting factor in terms of germination responsivity and there is no information about the physiological effects of other (supposedly) active butenolide compounds (the karrikins) present in the smoke. Therefore, we tested the typical and widely used 0.1 μM and 0.01 μM concentrations of KAR_1 _and 0.1 μM was chosen for the microarray experiment. However, due to the possibility of other potentially active compounds in smoke our primary interest was to assess smoke-water and KAR_1 _responsive genes and not to compare the two treatments (we could not assume that the molecular basis of smoke and KAR_1 _action is the same).

### Transcriptome analysis of smoke-water and KAR_1_-treated germinating kernels

**Figure 2:**
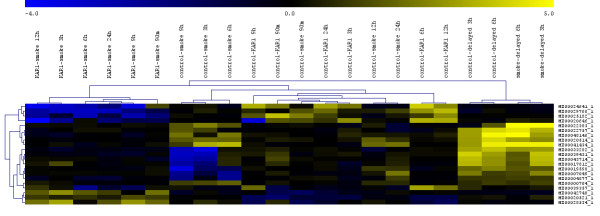
**Hierarchical clustering of data from the microarray analysis of gene expression in smoke- and KAR_1_-treated germinating maize kernels**. The data represents control vs. smoke, control vs. KAR_1_, control vs. smoke-treated for 3 h after a 3 h delay, control vs. smoke-treated for 6 h after a 3 h delay, and KAR_1 _vs. smoke comparisons. Samples with similar patterns of expression of the genes studied cluster together, as indicated by the dendrogram. The hierarchical clustering of the 21 genes that seemed to be the most relevant in all experiments is illustrated (expression changed in all experiments with fold-change ≥ 2, and at least in one experiments the corrected p-values < 0.1). Yellow indicates up-, and blue indicates downregulation.

In a previous study, we performed microarray analysis of smoke-water-induced germinating maize kernels (young seedlings) which had just entered phase III of germination characterised by rapid and pronounced water uptake [1]. In this study, to begin elucidating the molecular basis of smoke and KAR_1 _action during imbibition, before testa rupture, a detailed temporal analysis of gene expression under smoke and KAR_1 _exposure was conducted using microarrays. As the germination time course shows, the germination of maize is not perfectly synchronous, and the radicles/coleoptiles first appeared in the treated samples. We assumed that in the first 24 h of imbibition, before the first observation of testa rupture, the kernels are more homogenous in terms of developmental stage than later, and we chose early time points to collect the samples. Beyond 24 h, it is difficult, due to the increasing radicle emergence, to sample imbibed kernels in the same developmental stage. To further reduce the effect of differences in the germination stages, we used 90 embryos at every time point (15 embryos of six independent treatments). Embryos excised from kernels of the Mv255 maize strain treated with smoke-water (1:1000 dilution) and KAR_1 _(0.1 μM) solutions for 1.5, 3, 6, 9, 12 and 24 h were used for the experiment. We also investigated the changes in the transcription profile of embryos which were smoke-treated for 3 and 6 h, after a 3 h delay. In this experiment, control and smoke-treated samples were compared to samples which were imbibed in water for 3 h and then exposed to smoke-water for an additional 3 and 6 h. For the whole time-course experiment 68 independent microarray slides were used. The microarray data presented here have been deposited in the GEO database (http://www.ncbi.nlm.nih.gov/geo) under accession number GSE17484. Throughout the course of the experiments, only a narrow subset of genes were affected at all time points by the treatment. Figure 2 shows the expression pattern of 21 selected genes whose expression changed in all experiments with fold-change ≥ 2, with corrected p-values < 0.1 in at least two experiments (Additional File 1). Additional File 2 shows the expression patterns of all genes at all time points and comparisons which showed a fold-change ≥ 4 and a corrected p-value < 0.1 in at least one experiment. The full list of the genes with fold-change ≥ 2, their annotation and p-values in the different treatments and time points are available online as Additional Files. Genes with corrected p-value < 0.1 (regarded as significantly differentially expressed) are at the top of the list, separated with a red line.

The list of smoke-responsive genes (Figure 2; Additional Files 2 and 3) shows a significant overlap with our previous transcriptome data obtained from young smoke-treated maize seedlings 24 and 48 h after imbibition [1]. A sulfiredoxin-like protein gene (MZ00020514) and a LRR receptor kinase-like gene (MZ0000704) were upregulated, while the transcript abundance of calcineurin 9B-like gene (*CBL9*; MZ00043714) and an unknown gene (MZ00019598) with a tetratricopeptide repeat (*TTR*) sharply declined. In smoke-treated seeds, the most obvious changes were observed in the expression of the ubiquitin activating enzyme 1 (*UBE1*, MZ00041434) which was upregulated after 6 and 12 h.

To differentiate between imbibition/germination and the smoke-specific response, we compared the transcriptome of kernels imbibed in water for 3 h and additionally treated with smoke-water for 3 or 6 h with control ones in an independent experiment (Figure 2; Additional Files 2 and 4). This design allowed us to partially filter imbibition specific genes and narrow down the potential list of smoke specific genes. Interestingly, nearly the same expression pattern was obtained as in the time course study, with the overwhelming expression of *UBE1*, and surprisingly, the upregulation of the *CBL9*, *TTR *and two unknown genes (MZ00033282 and MZ00039431) which were downregulated in the time course experiment. The putative methylcrotonyl-CoA carboxylase (MZ00022757) showed unique and concerted upregulation when smoke was applied in delay.

**Figure 3:**
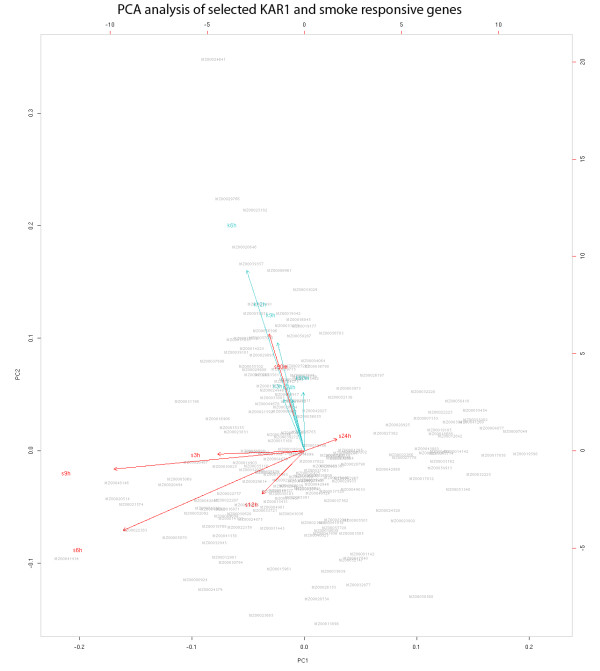
**Principal component analysis of the genes distinctly differentially expressed between smoke and KAR_1 _treatments**. Biplot representation of the principal component analysis. The figure shows the 199 genes at 1.5 h, 3 h, 6 h, 9 h, 12 h and 24 h that were distinctly differentially expressed (fold-change ≥ 4 and a corrected p-value < 0.1) in at least one experiment. All of the genes were plotted with respect to the first and second principal components and they are represented with a light gray text. The originally observed variables are plotted as red (smoke- related) and blue (KAR_1_-related) responsive arrows. The arrows represent the association of the measured variables (fold-change) with the samples in the visualization: the length and location are proportional to the variable loadings on the two first principal components. The analysis suggests that much of the variability and difference between the two gene sets can be attributed to the two different treatments (smoke and KAR_1_). Also see Additional File 10.

KAR_1 _treatment yielded a completely different gene expression pattern in comparison to smoke-treated samples (Figure 2, and 3; Additional File 2, 5 and 6). A senescence-associated protein-related gene (MZ00020646) was upregulated at all time points, except at 9 h, where a sharp decline in the expression was observed. A putative plastidic phosphate translocator-like protein 1 (MZ00025182) and a glycosyltransferase domain-containing gene (MZ00039357) was also constantly upregulated. The most notable gene, however, which was upregulated during the whole course of the experiment is a tonoplast intrinsic protein (*TIP3.1*), a member of the aquaporin family. Analysis of the microarray data obtained from comparison of the KAR_1_- and smoke-treated samples showed that the master genes *TIP3.1 *(MZ00024641), senescence-associated protein-related gene (MZ00020646) and S-adenosylmethionine-dependent methyltransferase (MZ00029766), which proved to be KAR_1_-responsive, were downregulated at almost all time points in smoke-treated plants (Figure 2; Additional Files 2 and 6). This latter gene functions in the flavonoid biosynthesis process and both smoke and KAR_1 _responsive gene lists were enriched in transcripts related to the phenylpropanoid pathway, although different gene sets were affected (Additional File 2). Smoke treatment induced the expression of anthranilate phosphoribosyltransferase (MZ00024875), flavanone 3-beta-hydroxylase (MZ00044256), flavonol glucosyltransferase (MZ00021805), flavonoid 3'-hydroxylase (MZ00021482) and *CYP71D *(MZ00029737), while the *CYP81E1/D8 *gene (MZ00004877) was downregulated. After KAR_1 _treatment, the transcript abundance of cinnamoyl-CoA reductase (MZ00036789), cinnamic acid 4-hydroxylase (MZ00036045) and anthranilate phosphoribosyltransferase (MZ00047824) were altered.

### Validation of microarray data by real-time quantitative RT-PCR

**Figure 4:**
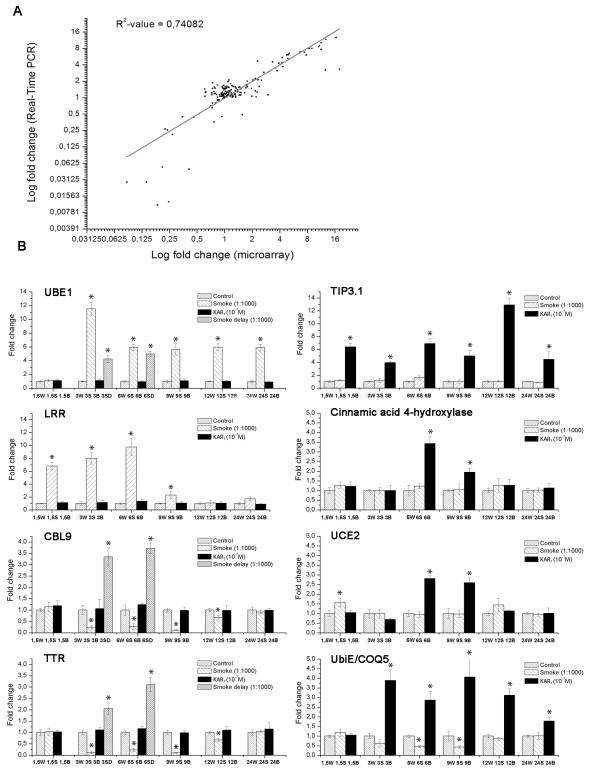
**Validation of microarray results via quantitative real-time PCR**. A, Quantitative real-time PCR analysis was performed for 14 genes under the same conditions and design used for microarray analysis. Real-time PCR data were obtained from three independent experiments with similar results, and four amplification reactions. Microarray data (least-square means) were plotted against data from qRT-PCR and fitted into a linear regression. Both x- and y-axes are shown in log2 scale. B, The biological variation of the expression (assessed by quantitative real-time PCR analysis) of 8 selected master genes are shown. Experimental design is as for Fig. 5A. Error bars represent standard deviation. Asterisks indicate significant difference (p < 0.05) from the control samples (statistical analysis was assessed by a t-test).

To validate the microarray results, the differential expression for selected genes was corroborated using qRT-PCR. Fourteen genes from various functional categories and displaying diverse expression profiles were chosen from among all differentially regulated genes. Despite the relatively high false discovery rate (FDR) in some cases (i.e. ~25% in 24 h smoke experiment and ~30% in 1.5 h and 3 h experiments, which were excluded from further analysis, or a moderate ~11% in the delayed experiments), the expression pattern observed in the microarray experiments was consistent with the genes analysed by real-time PCR (Figure 4A). The linear regression analysis showed a significant correlation between the two data sets, with R^2 ^= 0.74082. In addition, the expression response (fold change) of the selected key genes to smoke and KAR_1 _treatments showed little variation in the three independent experiments (Figure 4B).

### Gene Ontology analysis

**Figure 5:**
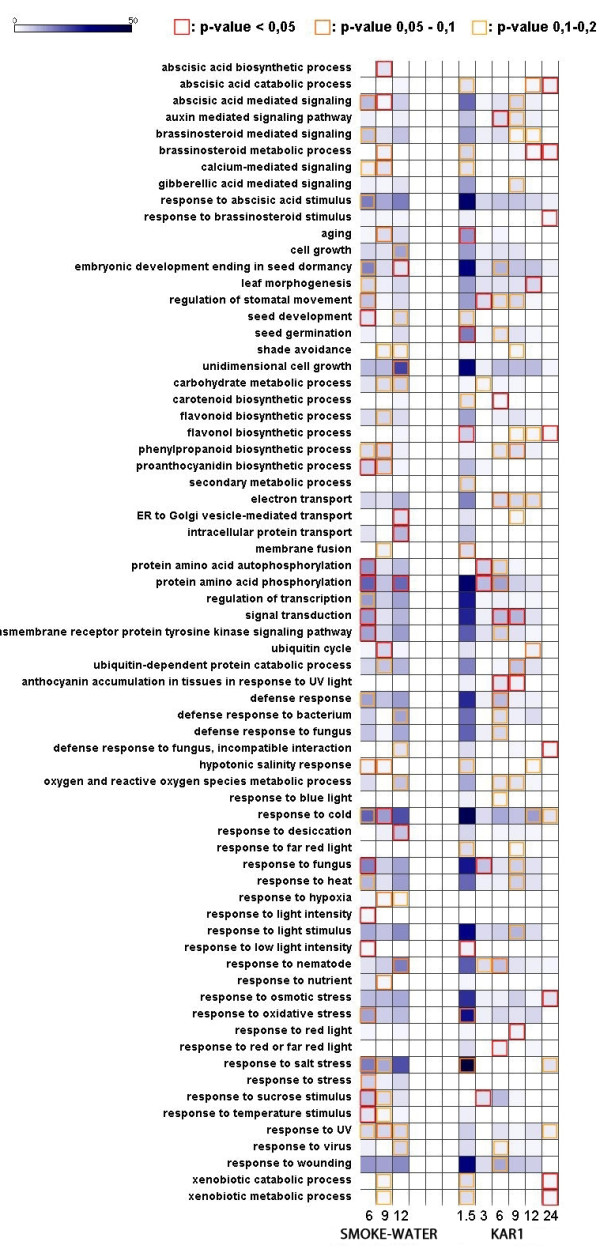
**The list of GO terms overrepresented in the group of genes up- and downregulated (fold change ≥2 and corrected p-value < 0.2)**. Data obtained from 1.5 h, 3 h and 24 h smoke-water treated samples are not included on the map. The frequency of each GO term was calculated [42] and multiplied by 100 to make the plotting easier on the heat map. Light colours indicate low representation; blue/deep blue colours show overrepresentation. Red squares: raw p-value < 0.05; orange squares: raw p-value of 0.05 - 0.1; yellow squares: raw p-value 0.1 - 0.2.

A stringent false discovery rate correction was applied to p-values when individual fold changes were studied but not when genes were studied in functional groups [14]. Genes up- or downregulated by ≥ 2-fold and with a corrected p-value < 0.2, due to smoke-water or KAR_1 _treatment, were associated with different Gene Ontology (GO) terms. Figure 5 shows the most highly represented GO terms and their raw p-values. For the entire Gene Ontology list and raw p-values, see Additional Files 7 and 8. The most pronounced GO terms following smoke-water or KAR_1 _treatment were quite similar, contrary to the fundamental differences in the up- and downregulated gene lists. The presence of stress-related genes were robust and extensive among the responses. A number of GO terms involved in cold, salt, heat, osmotic, fungus and other stress responses, light response ('response to low light intensity', 'response to light stimulus', 'response to blue light', 'shade avoidance') and ABA and brassinosteroid-responsiveness were enriched in both gene lists. Genes related to the phenylpropanoid metabolism and flavonoid biosynthesis were also represented in high number. As expected, genes related to ubiquitin-dependent protein catabolic process were abundant. Regarding hormone-related signatures, genes involved in the ABA stimulus were more prevalent, although auxin-mediated signalling pathway and brassinosteroid-related genes were also overrepresented. Gibberellin-related terms, however, were less frequent on the list. Growth and development related terms - 'seed germination', 'unidimensional cell growth', 'embryonic development ending in seed dormancy' were also abundant in both lists.

### Physiological response of germinating maize kernels to smoke-water and KAR_1_-treatment

**Figure 6:**
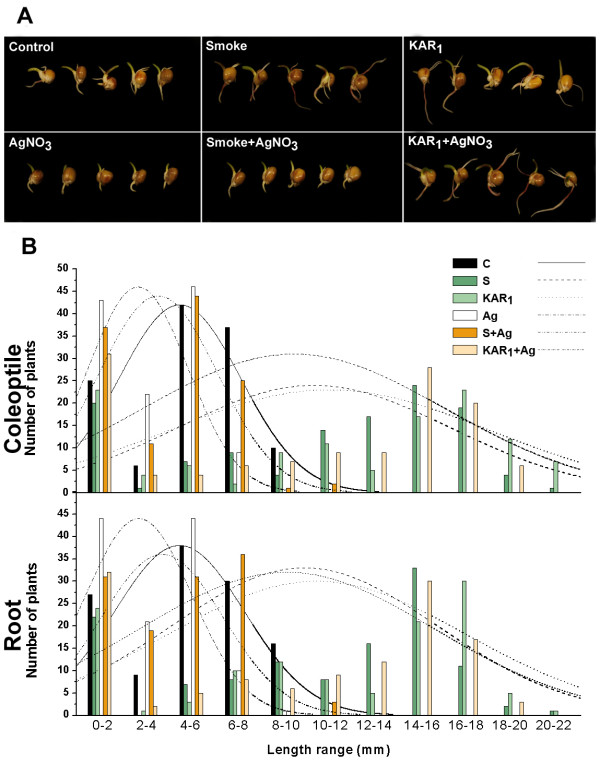
**Effect of smoke, KAR_1_, AgNO_3 _and their combinations on the seedling vigour of 5-d-old maize seedlings**. A, Typical phenotypes of the treated kernels. B, Frequency distribution of two growth variables (coleoptile and root length in mm) in control, smoke (1:1000 dilution), KAR_1 _(0.1 μM), AgNO_3 _(10 μM), smoke+AgNO_3_, KAR_1_+AgNO_3_. The data were grouped into bins as presented on the graph. For statistical analysis (see Additional File 9), the Mann-Whitney U-test was applied (R 2.9.0.) and p < 0.05 was regarded as significant (*n *= 120).

Experiments were carried out to determine the effect of smoke-water and KAR_1 _on the germination characteristics and growth parameters (root and coleoptile length) of 5-day-old maize seedlings and the potential interplay between smoke-water, KAR_1 _and aquaporin inhibitors (Figure 6). The kernels responded more explicitly to the different treatments so here we discuss the effect of smoke, KAR_1 _and different inhibitors on growth parameters only.

Smoke-water, applied as a 1:1000 (v/v) aqueous dilution of crude smoke extract, yielded significantly longer coleoptiles and roots compared to the control (Mann-Whitney + Shapiro tests, p < 10^-10^). Treatment of maize kernels with the 0.1 μM solution of KAR_1 _resulted in a very similar frequency distribution of coleoptile/root sizes as observed in smoke-treated kernels.

To support the findings of microarray data, and of TIP3.1 aquaporin playing a crucial role in KAR_1 _action, we conducted germination tests on KAR_1_-treated maize kernels (Figure 6). It was previously reported that KAR_1 _can alleviate the negative effect of aquaporin inhibitors like mercury chloride (HgCl_2_) and zinc chloride (ZnCl_2_) in tomato seedlings, indicating the possible involvement of aquaporins in KAR_1 _action [12]. We applied two known aquaporin inhibitors [15], HgCl_2 _and silver nitrate (AgNO_3_), on maize seedlings to determine the involvement of aquaporins in KAR_1 _action. Both treatments resulted in a reduction of the growth parameters of the seedlings, and the AgNO_3 _proved to be a stronger inhibitor (Mann-Whitney test, p < 10^-10^; Additional File 9). Treatment of the seedlings with a combination of KAR_1_, AgNO_3 _and HgCl_2 _showed an alleviation of the adverse effect of the AgNO_3 _and HgCl_2_, whereas simultaneous treatment with both smoke-water and AgNO_3 _or HgCl_2 _show no such reduction in the effect of AgNO_3 _and HgCl_2 _inhibition (Additional File 9). This effect of the KAR_1 _in combination with AgNO_3 _was demonstrated by the frequency distribution of the seedling shoot/root size which was not significantly different from the KAR_1_-treated plants (Figure 6; also see Additional File 9). Based on the assumption that AgNO_3 _treatment might interfere with ethylene perception, we also examined whether the KAR_1_-related transcriptome overlaps with ethylene-related gene expression patterns (genes regulated by endogenous basal level of ethylene and ethylene treatment in wild-type, ethylene insensitive mutant *etr1-1 *and the ethylene-constitutive mutant *ctr1-*1Arabidopsis plants [16]) and GO terms related to ethylene signalling or ethylene stimulus appeared in the list. Genes encoding almost every protein in the ethylene signal transduction pathway in Arabidopsis have also been found in maize previously, and ethylene signaling components also have similar biochemical functions [17]. The microarray data obtained from KAR_1_-treated plants showed no similarity with ethylene-related transcriptomes and no significant amount of GO terms related to these biological processes occurred in any of the gene lists (see Additional Files 7 or 8), suggesting that KAR_1_-treated seedlings may overcome the adverse effect of the silver ions not because of the involvement of ethylene-related events.

**Figure 7:**
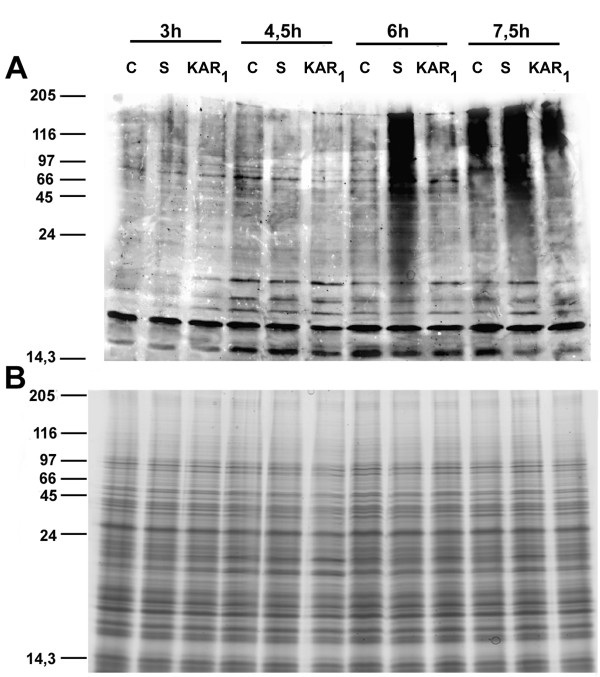
**SDS-PAGE and immunoblotting analysis**. Maize kernels were germinated in water (c, control) or were treated with smoke (s, 1:1000 dilution) or KAR_1 _(KAR_1_, 0.1 μM) for 3, 4.5, 6 and 7.5 h and proteins were extracted from the embryos (*n *= 15). The experiments were repeated three times with similar results. Molecular masses (kDa) of standard proteins are indicated on the left. A, Immunoblotting analysis with anti-ubiquitin antibody. B, Protein pattern obtained by SDS-PAGE and Coomassie Blue staining.

Microarray data indicated the possible involvement of ubiquitin-mediated protein degradation in smoke action. To further elucidate the findings revealed by the transcriptome data, the level of ubiquitinated proteins were examined using an anti-ubiquitin antibody. To demonstrate that smoke-water exposure has an effect on the ubiquitination process, we blotted the protein samples extracted from maize embryos after 3, 4.5, 6, and 7.5 h of smoke-water (1:1000 dilution) or KAR_1 _(0.1 μM) treatment onto PVDF membrane and treated it with antibodies raised against polyubiquitin (Figure 7). Comparing these samples with controls, and samples treated similarly with KAR_1_, it was apparent that smoke-treatment, and not KAR_1_, enhanced the ubiquitination of the proteins dramatically after 6 h. At 3 and 4.5 h, the level of ubiquitination was similarly low in both treatments, and at 7.5 h all the samples showed an increase in signal intensity, although in the smoke-treated samples the ubiquitinated proteins were more prevalent, suggesting that the smoke treatment resulted in accelerated ubiquitination. The proteins extracted from control and treated samples of the time course shared similar patterns, at least within the limits of SDS-PAGE and Coomassie staining techniques.

## Discussion

Over the past few years, the physiological effects of smoke and KAR_1 _treatments on seed germination have been investigated extensively, but only a few studies have discussed the deeper implications of smoke and KAR_1 _action [1,9,10]. This is the first report in which the effects of smoke and KAR_1_, during the first 24 h of imbibition, are assessed with respect to the molecular background of the phenomena. In agreement with previous investigations [11,1], our results show that smoke and KAR_1 _can accelerate germination, although their effect on seedling vigour is more pronounced.

The total transcriptome analysis revealed substantial differences in smoke- and KAR_1_-induced gene expression. The smoke-responsive gene list showed similarities with the transcriptome data obtained from young smoke-treated maize seedlings 24 and 48 h after imbibition [1]. The study revealed that 24 h after smoke-water treatment, the transcript abundance of sulfiredoxin-like protein (MZ00020514), the LRR receptor-like kinase (MZ00000704) and the *UBE1 *(MZ00041434) were the highest (with log fold change 4.79, 4.65 and 4.32, corrected p-value < 0.05, respectively), while the tetratricopeptide repeat containing protein (MZ00030105) was downregulated (log fold change -2.14, corrected p-value < 0.05) in the young seedlings, confirming that these genes could be the master genes in smoke action.

Smoke activated the ubiquitination-related UBE1 gene which catalyzes the first step in the ubiquitination reaction that targets proteins for degradation via the proteasome. Ubiquitin-mediated proteolysis plays a pivotal role in hormone synthesis, hormonal signalling cascades, plant developmental processes and stress responses (for review see [18]). There are many reports suggesting that ubiquitin-mediated proteolysis may also act upstream of the hormonal signalling cascades by regulating hormone biosynthesis, transport and perception and it is well established that hormonal cross-talk can occur at the level of proteolysis.

Smoke-water treatment yielded the formation of high molecular mass ubiquitin conjugates before the ubiquitination signal or degron appeared in the control and KAR_1_-treated kernels. The observed levels of ubiquitin conjugates, detected by immunoblotting using anti-ubiquitin antibodies, suggest an intense involvement of the ubiquitin-mediated proteolytic pathway during smoke-induced germination. It was previously shown that protein ubiquitination, and the subsequent protein degradation, is a key feature during seed germination [19]. In line with the expression data, the abundance of the *UBE1 *transcript reflects the functional activity of the enzyme. Presumably, application of smoke-water accelerates protein turnover and affects the assignment of proteins to be degraded by proteasomes and this eventually leads to the enhanced germination and seedling growth. Although the E2 and the more diverse E3 ligases are well characterized, the exact regulation of the E1 enzyme is poorly understood. Two ubiquitin activating enzyme clones from tobacco were induced after biotic stresses and stress hormones supporting the idea that the ubiquitin-proteasome system is activated as a stress response [20].

Smoke-water treatment resulted in the upregulation of other stress and developmental responsive genes. Plant peroxiredoxins (2-Cys-Prxs) are subject to substrate-mediated inactivation reversed by the smoke-induced sulfiredoxin, which suggests that the 2-Cys-Prx redox status and sulfiredoxin are part of a signalling mechanism participating in plant responses to oxidative stress [21]. Leucine rich repeats containing receptor-like kinases (LRR) comprise a large gene family which play important roles in plant growth and development as well as hormone and stress responses. The CBL9 calcium sensor, of which the expression was downregulated by smoke treatment, desensitizes ABA effects in seed germination since CBL9 functions as a negative regulator of ABA response in both seed germination and gene expression regulation in vegetative tissues [22]. Interestingly, *CBL9 *was upregulated when smoke was applied for 3 and 6 h after a 3 h delay. This unique expression pattern might be attributed to the partially-imbibed state of the seeds before smoke-water application which could result in a reduced uptake of the smoke compounds, or suggests that their expression is imbibition dependent.

KAR_1 _application resulted in the distinctive expression of TIP3.1 aquaporin. Plant aquaporins facilitate the transcellular movement of water and, in some cases, also the flux of small neutral solutes across a cellular membrane. It was shown that TIP expression is highly tissue specific and can be altered by hormones, especially ABA [23]. However, the function of each individual TIP isoform and the integrated function of TIPs under various physiological conditions remain elusive. One member of the TIP subfamily, the TIP1.1 showed increased expression in cold-stressed cotton cotyledons, suggesting a role in plant defence against environmental stresses by providing a suitable water balance under stress conditions [24]. The inhibition of water transport by gold and silver compounds [15,25] and mercury chloride have been reported in isolated vesicles from higher plants as well as in the intact root system [25]. However, ethylene perception can also be blocked by silver ions [26] and the interaction between the ethylene signal transduction and KAR_1 _cannot be ruled out. The similar trend, however, that was observed with mercury-KAR_1 _interaction shows that the TIP3.1 aquaporin plays an important role in KAR_1 _action, as previously suggested [12]. Furthermore, the KAR_1_-related transcriptome showed no similarity to ethylene-related transcriptomes [27] and no significant amount of GO terms related to ethylene occurred in any of the gene lists.

Contrary to the obvious differences in the primary action and in the lists of smoke- and KAR_1_-responsive genes, the treated kernels showed very similar germination responses after 5 d, with seemingly similar growth parameters. Furthermore, the genes can be classified into quite similar functional categories. It should be noted that due to the complexity of biological data-mining situations, in its current state, the analysis of large gene lists with the current gene set enrichment tools is still more of an exploratory data-mining procedure rather than a pure and exact statistical solution. The best analytical conclusions are made with the aid of the investigator's bio-knowledge, integrated annotation databases, computing algorithms and the enrichment p-values derived from statistical methods [28]. In our study, the occurrence of stress-related genes were robust and are extensive among the responses, especially cold, heat and biotic stresses, although the p-values calculated showed less significant results. Salt, osmotic and other stress-related terms were also abundant, as observed in the early post-germinative phase of smoke-treated maize seedlings [1] or in smoke-treated achenes of Grand Rapids lettuce [9]. Genes involved in the light response were also predominant in both lists suggesting a presumptive involvement of light signalling in smoke action. This assumption is in accordance with the finding that smoke and KAR_1 _can replace the light requirement of the germination of *Lactuca sativa *cv. Grand Rapids achenes [6]. KAR_1_-stimulation of Arabidopsis germination is light-dependent and reversible by far-red light exposure, suggesting a possible involvement of light signalling in KAR_1 _action [10]. However, it can be considered that the over-representation of stress- and light-response-related terms in the GO lists may indicate that the active constituents are perceived and the signal is mediated in a similar way as environmental stress signals and therefore general stress-related pathway integrators could play a crucial role in smoke and KAR_1 _action. The CBL9 is a good example of this type of signal integrator, since it mediates the cross-talk between hormones and its expression is affected by stress [22]. Given the diversity of LRR kinases and more than 700 F-box proteins present in the Arabidopsis genome, it is especially intriguing to consider the extensive possibilities for small-ligand-based signal perception mediated by these potential receptors and signal transduction pathways [29]. These pathways are also the source of the immense complexity of plant biochemicals, meaning that a host of additional 'growth regulators' might lie undiscovered [30]. The remarkable occurrence of phenylpropanoid pathway related genes for both treatments may suggest the importance of flavonoids in the smoke and KAR_1 _action. Apart from their function in the *Rhizobium*-legume and in different plant-soil pathogen interactions, flavonoids have been implicated in the modulation of developmental processes as diverse as auxin transport, pollen germination, root hair growth, allelopathic responses and in systemic acquired resistance [31]. The induction of several key enzymes of the phenylpropanoid pathway raises the question whether KAR_1 _and other active compounds are metabolized in plants forming a so far unknown class of growth regulators [4].

The obvious differences between the smoke- and KAR_1_-responsive gene lists clearly indicates the interaction of other germination-active cues in the smoke which together form the physiological response towards smoke treatment. In addition to the KAR_1 _used in this study, at least five other active butenolides are known to be present in smoke [5,10] and other active compounds are suspected to exist [4,7]. It was previously reported that smoke-water has a 'dual regulatory' effect on germination, since high concentrations of smoke-water were shown to inhibit germination, whereas lower concentrations had a promotory effect [7]. The assumption that inhibitory cues may also be present in the smoke was recently supported by the isolation of a related butenolide, 3,4,5-trimethylfuran-2(5*H*)-one, that results in an inhibitory effect on the germination of lettuce achenes [8]. Considering the assumption that the smoke effect (and the effect of the active promoter compound) is modulated by the presence of the inhibitory and other promoter compound(s) (e.g. KAR_2_-KAR_6_), we applied typical smoke-water and KAR_1 _concentrations which are regarded as equivalent in terms of their observed physiological activity. KAR_1 _is equally and uniformly active over a wide concentration range between 10^-4^-10^-9 ^M [4]. Smoke-water (a standard batch used in our laboratories) was used in diluted form between 1:10 - 1:2000, with the higher concentrations having an inhibitory effect [7], and the most widely used effective dilutions being between 1:500 - 1: 2000 in our previous studies. We showed that the undiluted form contains the inhibitory compound in high concentration and by dilution of the smoke-water the inhibitory effect can be diminished. The effect of smoke-water, however, depends on the production of the smoke-water and also depends on the species used for the germination assay. In the present study, our results showed that the concentrations used previously in germination studies are not equivalent in terms of the expression pattern induced. Our results, together with earlier findings, clearly indicate that the array of compounds present in the smoke results in distinctly different effects on the gene expression in germinating maize kernels in comparison to that observed with the treatment of KAR_1 _alone. This is to be expected considering the number of active compounds found in smoke and smoke-water. The presence of more potentially active compounds in smoke, the concentration-dependent activity of the inhibitory compound, and their possible interactions implies that no two batches of smoke can be regarded as exactly the same, or the presence of the active compounds should be monitored in parallel. However, it should be noted that the list of potential smoke-responsive genes shows a considerable overlap with the expression pattern of embryos in the early post-germinative stage treated with a completely different batch (batch No. "1") of smoke-water [1]. We cannot necessarily draw the conclusion that different smoke batches have the same effect on the expression pattern, as this would require a more detailed investigation.

The positive and negative germination cues represent a diverse suite of chemical signals provided by the environment to signal germination. These compounds may fine-tune the germination response, and it may be possible that together they would compose a distinct signal required by fire-prone species to accurately locate their germination niche. It is of great interest that the tri-substituted but-2-enolide ring is a common structural feature of these butenolide compounds. The molecular basis of the effect of smoke may be related to the diverse binding affinity of the active compounds to the proposed receptor and the consequent effects exerted on the changes of gene expression patterns. Conducting in-depth molecular biology studies on the interaction of these compounds will definitely add a further dimension to the emerging picture on the effect of smoke on seed germination in fire-prone environments.

## Conclusions

In conclusion, accelerated protein degradation or induction of the TIP3.1 aquaporin are key features of smoke and KAR_1 _action. Considering all the knowledge accumulated to date in terms of smoke action we can assume that these physiological events represent only the 'tip of the iceberg' and these can be regarded as the executers of smoke and KAR_1 _action. As far as the nature of smoke and KAR_1 _perception is concerned, it is highly possible that the smoke 'signal' is perceived by a receptor that is shared with the signal transduction system implied in perceiving environmental cues (especially stresses and light), or some kind of specialized receptor exists in fire-prone plant species which diverged from a more general one present in a common ancestor, and also found in the non fire-prone plants allowing for a somewhat weaker but still significant response. These major integrators of environmental signals, stress and hormone responses, could be potential targets for future research.

## Methods

### Plant Material, growth conditions and germination tests

For the germination tests, microarray studies and western blotting experiments, kernels (seeds) of *Zea mays *L. Mv255 strain were used. The kernels were stored in refrigerators at 4°C in paper bags until use. Decontamination was done in 3% sodium hypochlorite containing Tween 20 and 70% EtOH (10 min each). In the germination time course tests, each treatment consisted of four independent experiments with three biological replicates (30 kernels in each). The kernels were placed in 90 mm Petri dishes on tissue paper moistened with water (control), 1:1000 or 1:2000 (v/v) dilution of smoke-water, 0.1 or 0.01 μM KAR_1_, and allowed to germinate in a controlled environmental chamber (25°C, 80% RH, and 100 μmol m^-2 ^s^-1 ^light intensity). Germinated kernels (kernels with visible roots and coleoptile) were scored every day at the same time for 10 d. In the vigour tests, each treatment consisted of two independent experiments with two biological replicates (30 kernels in each). Batches of kernels were submerged for 1 h into 20 mL water (control), 1:1000 (v/v) dilution of smoke-water, 0.1 μM KAR1, 30 μM AgNO_3 _and their combinations (smoke-water+AgNO_3_, KAR_1_+AgNO_3_) at the same concentrations. Thereafter, the kernels were placed and incubated for 5 d under the same temperature and light regime as described earlier. For statistical analysis, the Mann-Whitney U-test was applied with the R 2.9.0 software [33] and p < 0.05 values were regarded as significant.

### Preparation and GC-MS analysis of smoke-water

The smoke-water (batch No. "2") was prepared from burnt *Themeda triandra *Forssk. (Poaceae), according to the method outlined in Baxter et al. [32]. The butenolide, 3-methyl-2*H*-furo[2,3-*c*]pyran-2-one (KAR_1_), was synthesised from pyromeconic acid according to Flematti et al. [33]. The inhibitory compound was synthesized according to Light et al. [8]. The GC-MS analysis of KAR_1 _and the inhibitory compound content of smoke-water was carried out with slight modifications using a Shimadzu Model GCMS-QP2010 system (Shimadzu) fitted with an SP-2380 capillary column (30 m · 0.25 mm I.D., df = 0.20 lm; Supelco/Sigma-Aldrich) according to Flematti et al. [5] and Light et al. [8], respectively. Peaks were identified according to the retention times and mass spectra of standards.

### RNA isolation

For RNA isolation from control, smoke- (1:1000) or KAR_1_-treated (0.1 μM) kernels, identical conditions were applied as for the vigour tests and embryos were harvested 1.5, 3, 6, 9, 12, 24 and 27 h after placing them in the Petri dishes. At 24 h, only the kernels with no testa rupture were selected for further experiments. In an additional experiment, control and smoke-treated samples were compared to samples which were imbibed in water for 3 h and then exposed to smoke-water for an additional 3 or 6 h. Individual kernels (15) from each of six independent biological replicates were chosen and then the embryo axes (without scutellum) were excised with a scalpel and frozen immediately in liquid nitrogen in batches. Total RNA was isolated using TRIzol reagent (Invitrogen) and cleaned up with RNeasy Plant Mini Kit (Qiagen). The RNA Integrity Number (RIN) of the samples was determined using the Agilent BioAnalyzer. Only samples with a RIN ≥ 8 were considered for further analysis.

### Microarray platform, labelling, hybridization and image acquisition

The microarray study was performed according to Soós et al. [1] with a few modifications. The experimental design was generally based on the instructions of Kendziorski et al. [35] and Dobbin et al. [36]. The RNA samples of six parallel and independent experiments (each containing 15 kernels) were used and an equal amount of the aaRNA samples (see later) were pooled (RNA of 90 individual embryos in total) from the six experiments. Three technical replicates were applied at each time point for the microarray analysis. Microarray slides with ~46K features were manufactured by the University of Arizona Maize Array Project (http://www.maizearray.org). The detailed array annotation, composition and the experimental procedures followed in this work can be found at the Internet site. In brief, 400 ng total RNA was amplified and aminoallyl-UTP was incorporated using TargetAmp Kit (Epicentre) and the resulting aaRNA was labelled with Cy3 and Cy5 (Amersham). The dye-labelled probes were then cleaned up (Qiagen), mixed with the corresponding samples, concentrated, resuspended in the hybridization solution and incubated at 42°C overnight in a hybridization oven. Finally, the slides were washed with different concentrations of SSC at room temperature.

The detailed description of the various hybridizations is the following: All the samples from control experiments were compared to samples from smoke-treated experiments with 3 technical replicates, totalling 21 microarray slides. The samples from control experiments were also compared to samples from KAR_1_-treated experiment with 3 technical replicates, totalling 18 microarray slides. The samples from KAR_1_-treated experiments were compared to samples from smoke-treated experiments with 3 technical replicates (except in the case of the 3 h samples, where only 2 technical replicates were used), totalling an additional 17 slides. Finally, 3 and 6 h control samples were compared to the 3 and 6 h delayed samples, and also 3 and 6 h smoke-treated samples were compared to the 3 and 6 h delayed samples with 3 technical replicates, totalling 12 slides.

Scanning was performed using an Amersham Typhoon Trio+ with default settings. The detection of signal intensities and the grid adjustment were accomplished with ArrayVision v8.0 (Amersham). The intensity value of each spot and background region, multiplied by its area was used as signal intensity for further analysis.

### Microarray data normalization and analysis

Raw intensity data were imported into the R2.9.0 [34], after pre-processing it with custom made Perl scripts. Further analysis was carried out using the LIMMA [37] package of BIOCONDUCTOR [38]. Background correction was done using the normexp method [39]. Normalization of data within arrays was done using the loess method [40]. To normalize the data between arrays the quantile method was used [41]. The microarray data for each gene were fitted to a simple control versus treatment linear model at each time point/comparison, and statistics were generated using the lmFit and eBayes functions [42] of the LIMMA package. The p-values were adjusted for multiple testing using the method of Benjamini and Hochberg [43]. Genes with fold-change ≥ 2 and a corrected p-value < 0.1 were considered as differentially expressed. The microarray data presented here have been deposited in the GEO database (http://www.ncbi.nlm.nih.gov/geo; accession number GSE17484). The dataset contains the expression data obtained from kernels exposed to smoke for 27 h which is not discussed here.

### Gene Ontology analysis

For Gene Ontology analysis, less stringent conditions (corrected p-value < 0.2) were applied as for the expression analysis [14]. Based on the available chip annotation supplied by the University of Arizona Maize Array Project (http://www.maizearray.org), the genes were assigned into the available Gene Ontology categories [44], and the significant over-representation of particular categories in the combined up- and downregulated gene sets were determined. For the analysis we used the GeneMerge software [45], which uses the hypergeometric distribution for obtaining the rank scores for the overrepresentation of the studied gene sets (the upregulated genes) compared to the population gene sets (the full set of maize genes). We also modified the GeneMerge script, to reduce the large number of IO operations and the running time of a given analysis.

### Real-time PCR

The DNase I (Qiagen) treated mRNA samples (200 ng) extracted from three independent biological replicates (15 kernels each) were reverse transcribed with SuperScript III reverse transcriptase (Invitrogen). Real-time PCR was performed with Applied Biosystems 7500 using SYBR Green detection chemistry (Applied Biosystems) and gene-specific primers. Real-time PCR data were obtained from three independent experiments (not the same used for microarray analysis), and the reactions were performed in quadruplicate. Prior to the real-time PCR experiment, the applicability of the maize actin (AY103587) endogenous control was checked and we found that its expression in the embryo was unstable during the first 6 h of the imbibition. Based on our microarray data and the recommended reference gene list of Czechowski et al. [46], we used a putative RNA-binding protein gene (Accession no.: BT040552) as an internal control. The relative ratio of threshold cycle (Ct) values between the endogenous control and the specific gene were calculated for each sample. The validation procedure was performed with the same experimental design (all time points and treatments) as for microarray analysis using the following genes (Operon oligo identifiers are in brackets): UBE1 (MZ00041434), cytochrome P450 (MZ00022704), CBL9 (MZ00043714), unknown (MZ00039967), LRR receptor-like kinase (MZ00000704), RING3 protein (MZ00007049), ZmTIP3-1 (MZ00024641), TTR-containing gene (TTR; MZ00019598), sulfiredoxin (MZ00020514), CYP81E1/D8 (MZ00004877), cinnamic acid 4-hydroxylase (MZ00036045), YHVR-like protein (MZ00041687), S-adenosylmethionine-dependent methyltransferase/methyltransferase (UbiE/COQ5; MZ00029766) and ubiquitin-conjugating enzyme (UCE2; MZ00041882).

### Protein extraction and immunoblotting analysis

Kernels of maize strain Mv255 were raised under the same conditions as described above for RNA extraction. For protein isolation, embryo axes (without scutellum) from control, smoke-water (1:1000) and KAR_1_-treated (0.1 μM) samples were harvested after 3, 4.5, 6 and 7.5 h of treatment from three biological replicates. Fifteen embryos from each replicate were collected and ground to a fine powder in a mortar with liquid nitrogen. Each sample was resuspended in 500 μL extraction buffer (containing 2% (v/v) SDS, 5% (v/v) glycerol, and 2.5% (v/v) mercaptoethanol in 50 mM Tris-HCl, pH 6.8) with protease inhibitor cocktail (Sigma). The suspensions were thoroughly vortexed, then boiled for 10 min and centrifuged at 14000 g for 15 min. Total protein quantification was carried out following the Bradford's procedure.

From each experimental condition, 5 μg of protein was separated using SDS-PAGE on 12% acrylamide gels. Protein molecular weight standards in the range of 6.5-205 kDa (Amersham) were used as standards. The gels were then stained with Coomassie Brilliant Blue G-250 (BioRad) or the proteins were blotted onto low-fluorescent Hybond-LFP PVDF membranes (Amersham). The blotted membranes were blocked with 5% BSA/TBS-T for 1 h at room temperature, and probed overnight with Ub(6C1) mouse anti-ubiquitin antibody (Santa Cruz) in 1:2000 dilution. The immune complexes were detected using Cy3-labeled goat-anti-mouse IgG antibody in 1:4000 dilution (ECL Plex System, Amersham) and images were captured with Amersham Typhoon Trio+ scanner.

## Authors' contributions

VS designed the experiments, conducted the germination tests, the microarray experiments, the immunoblot analysis and wrote the paper, ES designed the experiments, analysed the germination tests and microarray data and wrote the paper, AJ designed the experiments, analysed the germination tests and microarray data and wrote the paper, MEL isolated the compounds and wrote the paper, GS and JT carried out the GC-MS measurements, LK synthesised the compounds, JVS assisted in experimental design and wrote the paper, EB designed the experiments, analysed the data and wrote the paper. All authors read and approved the manuscript.

## Supplementary Material

Additional file 1 **The fold change and corrected p-values of the 21 selected smoke and KAR_1 _responsive genes at all experiments and time points**. Click here for file

Additional file 2 **Hierarchical clustering of data from the microarray analysis of gene expression in smoke- and KAR_1_-treated germinating maize kernels**. The data represents control vs. smoke, control vs. KAR_1_, control vs. smoke-treated for 3 h after a 3 h delay, control vs. smoke-treated for 6 h delay after a 3 h delay, and KAR_1 _vs. smoke comparisons. Samples with similar patterns of expression of the genes studied cluster together, as indicated by the dendrogram. The hierarchical clustering of 212 genes that were distinctly differentially expressed (fold-change ≥ 4 and a corrected p-value < 0.1 in at least one experiment) is illustrated. Yellow indicates up-, blue indicates downregulation. Click here for file

Additional file 3 **The full list of the genes that were differentially expressed at any time point after smoke exposure**. Annotations and p-values are indicated. Genes with corrected p-value < 0.1 (regarded as significantly differentially expressed) are at the top of the list, separated with a red line. Click here for file

Additional file 4 **The full list of the genes that were differentially expressed at any time point when smoke-water was applied for 3 and 6 h after a 3 h delay. Annotations and p-values are indicated**. Genes with corrected p-value < 0.1 (regarded as significantly differentially expressed) are at the top of the list, separated with a red line. Click here for file

Additional file 5 **The full list of the genes that were differentially expressed at any time point after KAR_1 _exposure**. Annotations and p-values are indicated. Genes with corrected p-value < 0.1 (regarded as significantly differentially expressed) are at the top of the list, separated with a red line. Click here for file

Additional file 6 **The full list of the genes that were differentially expressed at any time point in the KAR_1 _vs. smoke comparison**. Annotations and p-values are indicated. Genes with corrected p-value < 0.1 (regarded as significantly differentially expressed) are at the top of the list, separated with a red line. Click here for file

Additional file 7 **List of GO terms related to smoke action**. Click here for file

Additional file 8 **List of GO terms related to KAR_1 _action**. Click here for file

Additional file 9 **Statistical analysis of the germination experiments**. For statistical analysis, the Mann-Whitney U-test was applied with the R 2.9.0 software and p < 0.05 values were regarded as significant (*n *= 120). Only kernels with both roots and coleoptile were regarded as germinated. Click here for file

Additional file 10 **Details of the principal component analysis of the KAR_1 _and smoke treatments on 199 differentially expressed genes at each time point**. Click here for file
