# Supplementary figures and images for: Transcriptome analysis of germinating maize kernels exposed to smoke-water and the active compound KAR1
Source: BMC Plant Biol. 2010 Nov 2;10:236. doi: 10.1186/1471-2229-10-236 (PMC3095319; doi:10.1186/1471-2229-10-236)

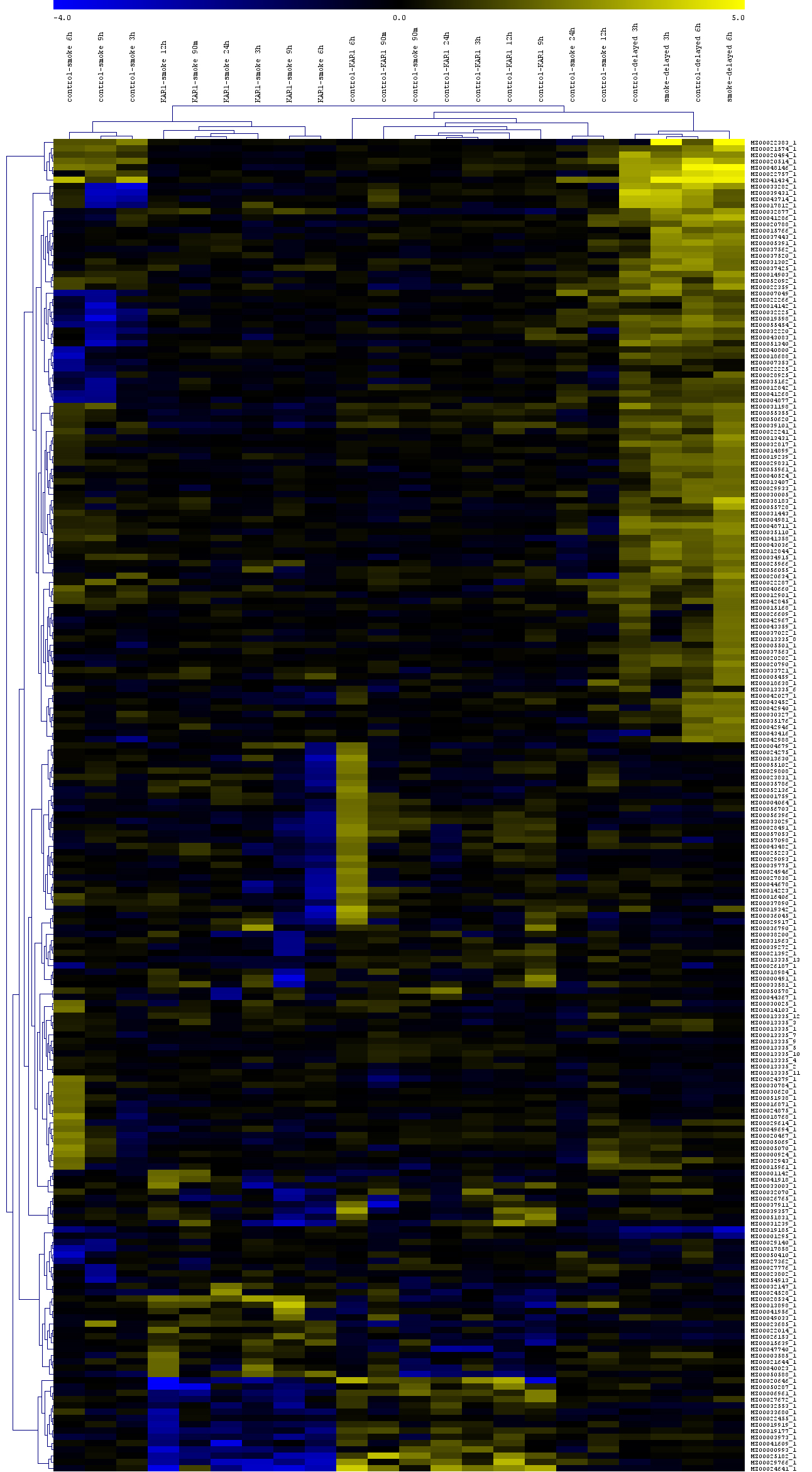

Supplement: Additional file 2 — Hierarchical clustering of data from the microarray analysis of gene expression in smoke- and KAR1-treated germinating maize kernels. The data represents control vs. smoke, control vs. KAR1, control vs. smoke-treated for 3 h after a 3 h delay, control vs. smoke-treated for 6 h delay after a 3 h delay, and KAR1 vs. smoke comparisons. Samples with similar patterns of expression of the genes studied cluster together, as indicated by the dendrogram. The hierarchical clustering of 212 genes that were distinctly differentially expressed (fold-change ≥ 4 and a corrected p-value < 0.1 in at least one experiment) is illustrated. Yellow indicates up-, blue indicates downregulation. [file 1471-2229-10-236-S2.JPEG]
